# Geographic variation in the response of *Culex pipiens* life history traits to temperature

**DOI:** 10.1186/s13071-016-1402-z

**Published:** 2016-02-29

**Authors:** Jordan E. Ruybal, Laura D. Kramer, A. Marm Kilpatrick

**Affiliations:** Department Ecology and Evolutionary Biology, University of California, Santa Cruz, CA 95064 USA; New York State Department of Health, Wadsworth Center, Slingerlands, NY 12159 USA; State University of New York at Albany, Albany, NY USA

**Keywords:** Climate change, West Nile virus, Thermal response, Vector-borne disease, Local adaptation, Reaction norm, Chikungunya virus, Dengue

## Abstract

**Background:**

Climate change is predicted to alter the transmission of many vector-borne pathogens. The quantitative impact of climate change is usually estimated by measuring the temperature-performance relationships for a single population of vectors, and then mapping this relationship across a range of temperatures or locations. However, life history traits of different populations often differ significantly. Specifically, performance across a range of temperatures is likely to vary due to local adaptation to temperature and other factors. This variation can cause spatial variation in pathogen transmission and will influence the impact of climate change on the transmission of vector-borne pathogens.

**Methods:**

We quantified variation in life history traits for four populations of *Culex pipiens* (Linnaeus) mosquitoes. The populations were distributed along altitudinal and latitudinal gradients in the eastern United States that spanned ~3 °C in mean summer temperature, which is similar to the magnitude of global warming expected in the next 3–5 decades. We measured larval and adult survival, development rate, and biting rate at six temperatures between 16 and 35 °C, in a common garden experiment.

**Results:**

Temperature had strong and consistent non-linear effects on all four life history traits for all four populations. Adult female development time decreased monotonically with increasing temperature, with the largest decrease at cold temperatures. Daily juvenile and adult female survival also decreased with increasing temperature, but the largest decrease occurred at higher temperatures. There was significant among-population variation in the thermal response curves for the four life history traits across the four populations, with larval survival, adult survival, and development rate varying up to 45, 79, and 84 % among populations, respectively. However, variation was not correlated with local temperatures and thus did not support the local thermal adaptation hypothesis.

**Conclusion:**

These results suggest that the impact of climate change on vector-borne disease will be more variable than previous predictions, and our data provide an estimate of this uncertainty. In addition, the variation among populations that we observed will shape the response of vectors to changing climates.

**Electronic supplementary material:**

The online version of this article (doi:10.1186/s13071-016-1402-z) contains supplementary material, which is available to authorized users.

## Background

The impact of climate change on the transmission of vector-borne diseases is a hotly debated topic [1–6]. Early predictions suggested that climate change would increase the global burden of tropical diseases, such as malaria, as temperate regions warmed [7]. However, other researchers have argued that warming will also cause a decrease in transmission in some tropical regions which will become too hot, and this will result in a geographic shift in distribution but little change in overall disease burden [8, 9]. Further, many have argued that changes in other factors such as socioeconomic development, land use, drug treatment and bed-net use will be more important than climate change in determining disease incidence, and that transmission will be limited in temperate regions by public health systems and highly developed living conditions (e.g. screened windows and air conditioning) [10].

An issue that has received far less attention, despite its potential impact, is variability in the response of vectors to temperature [8, 11]. Determining the extent of variation in temperature responses, is necessary to predict current and future spatial variation in transmission of tropical vector-borne diseases such as dengue virus and malaria, and to determine the extent of uncertainty in model predictions [11]. If variation among vector populations exists, and the variation is strongly correlated with local thermal regimes (as would be expected if adaptation to local temperatures were the strongest driver), then this variation could be incorporated into model predictions. However, organisms are simultaneously under a diverse set of selective pressures, and selection on life history traits from other factors, as well as drift, could result in unpredictable variation [12, 13]. Several studies have found either inverse or counter-gradient variation (a phenomenon in which variation in genotypes counteracts environmental influences across a gradient such that phenotypic variation is diminished) along temperature gradients [14], or significant variation, but little evidence of local thermal adaptation [15, 16]. If variation among populations in the response to temperature is substantial, but idiosyncratic, then predictions of the impact of climate change will be far less accurate, and models should incorporate this additional source of uncertainty into the predictions.

Only a handful of studies have been conducted on local adaptation to temperature in mosquito vectors. In *Anopheles gambiae*, an important vector of malaria in Africa, populations along aridity and latitudinal clines in Cameroon and Nigeria had increased frequencies of a genetic trait (the 2La chromosomal inversion) that confers increased heat and desiccation tolerance [17–20]. In contrast, a study of two populations of *Culex tarsalis* in California did not find variation in life history traits that correlated with local temperatures [21]. Clearly, additional studies are needed of variation in vector traits that influence transmission from multiple populations along temperature gradients [11].

We examined spatial variation in life history traits of *Culex pipiens* mosquitoes along altitudinal and latitudinal gradients to determine the extent of local thermal adaptation. *Cx. pipiens* is the primary enzootic (bird-to-bird) and bridge (bird-to-human) mosquito vector of West Nile Virus (WNV) and other arboviruses in urban and residential areas of North America north of approximately 36° latitude [22–26], and a vector of WNV and Usutu virus in Europe [26–28]. WNV is a significant public health issue in North America, with ~2.8 million human infections, >20,000 cases of encephalitis and 1902 deaths since it was introduced in 1999 [29, 30]. Additionally, WNV has also killed millions of birds and caused regional declines in some species of up to 50 % [31–33]. The wide geographic distribution of *Cx. pipiens* and its importance in transmitting several arboviruses makes it a useful model species to examine the extent of adaptation to local thermal regimes.

We conducted a common garden study of *Cx. pipiens* mosquitoes from four populations along altitudinal and latitudinal gradients with average summer temperatures differing by 2–3 °C (Additional file 1: Figure S1). This variation in temperature is similar to that predicted to occur over the next few decades due to anthropogenic climate change [34]. We combined altitudinal and latitudinal gradients to generate temperature gradients with different confounding variables (e.g. day length, atmospheric pressure, etc.). We measured four life history traits: larval and adult survival, development rate, and biting rate, across a range of temperatures that spanned the seasonal climate of these four populations to characterize their performance along a thermal gradient. We hypothesized that high temperature populations (lower latitude and elevation) would experience selection for faster development rate, and increased survival at hotter temperatures, whereas colder populations would exhibit the opposite tradeoff [35, 36].

## Methods

### Study sites

**Fig. 1:**
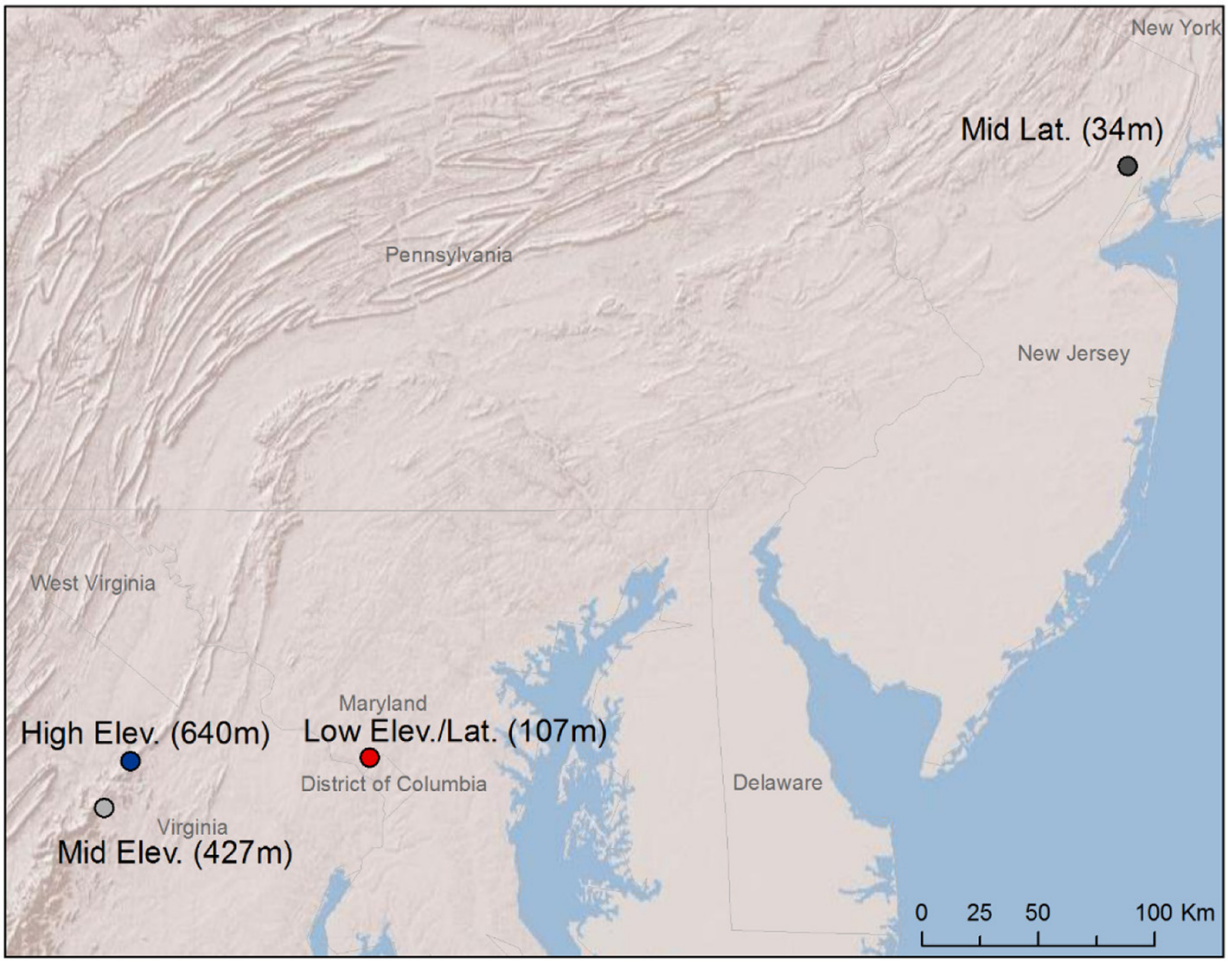
Map of study sites. Site names describe the latitude and/or elevation for each population of *Cx. pipiens* relative to the low elevation/latitude population and the elevation height is in parentheses

We collected an average of 24.5 (6, 15, 32, and 45 rafts, respectively from the coolest to the warmest site) *Cx. pipiens* egg rafts at each of four sites between July 25 and July 28, 2011 (Fig. 1b). Site names describe the latitude and/or elevation for each population relative to the low elevation/latitude population (Fig. 1b). *Culex pipiens* hybridize with *Culex quinquefasciatus* across a wide latitudinal band of North America [37, 38], and although previous genetic analyses in the study area found little evidence of *Culex quinquefasciatus* ancestry in these populations [39], introgression of selected alleles from this tropical species could influence the response of populations to temperature.

### Rearing and handling

All field collected eggs were hatched at 25 °C (±2 °C) under a photoperiod of 14:10 hrs (L:D), and larvae were morphologically identified to the *Cx. pipiens* complex using published keys [40]. Larvae were reared at 25 °C in groups of ~200 in Sterilite® plastic trays (27.9 cm L × 16.8 cm W × 7.0 cm H), filled with 1 liter of deionized water. Larvae were fed a finely ground 1:1:1 mixture of MP® Liver Powder Bovine, Kaytee Koi’s Choice® Premium Fish Food, & Small World® alfalfa rabbit feed. Adults were transferred to a 30.5 cm^3^ aluminum mesh collapsible cage (BioQuip) and held at 25 °C. Five day-old mosquitoes were deprived of sucrose, but not water, overnight (~12–15 h) and then fed a mixture of defibrinated chicken blood (Rockland Immunochemicals) plus a final concentration of 2.5 % sucrose and 1 % ATP, warmed at 37 °C for 5 min in a water bath. Females oviposited en masse and we collected an average of 61 egg rafts (20–85 rafts per population). Larvae from each population were pooled together, and 600 larvae from each population were placed into three trays (200 larvae each), except for the high elevation population which only had one tray with 200 larvae. This first laboratory–raised generation of offspring was used for measuring the response of each population to variation in temperature.

We used six fixed temperatures (one per incubator) that spanned the minimum and maximum summer temperatures experienced across study populations: 16, 20, 24, 27, 31, and 35 °C. Relative humidity (70 ± 10 %) and photoperiod (16:8 h. (L:D)) were held constant in all five incubators and larvae (and adults) were maintained as described above. Each day dead larvae and pupae were counted and removed. However, counts revealed that larvae had also disappeared due to cannibalism. Pupae from replicate trays were combined and transferred to a single emergence jar (BioQuip). The number of emerged males and females was counted daily and adults were immediately transferred to one-gallon cardboard containers with mesh tops. At 35 °C no larvae survived to become adults. As a result, we used the remaining larvae from the initial rearing temperature (25 °C) to measure adult daily survival and biting rate at 35 °C.

Adult mosquitoes were fed ad libitum Domino® sugar cubes and water. We monitored adult mortality by inspecting each cage daily and counted and removed dead adults. Females had constant access to an oviposition site-a small cup filled with deionized water. Blood meals were offered every 2 days for populations in the 35 & 20 °C incubators, every 3 days for populations in the 24, 27 and 31 °C incubators, and every 4 days for populations in the 16 °C incubator. We offered blood meals at different intervals in the different temperature treatments due to limited personnel and logistical difficulties, but this reduced our power to detect differences among populations, and should be avoided in future studies, if possible.

### Statistical analysis

All statistical analyses were done in R v3.1.1 (R Development Core Team 2012). We used generalized linear models to quantify the effects of population and temperature on female development time, larval emergence, larval cannibalism, and larval survival, and included two-way interactions between population and temperature to allow for the effect of temperature to vary among populations. We included linear and quadratic terms for temperature because residuals from linear models showed obvious evidence of nonlinearity. We calculated the Q_10_ temperature coefficient for larval development rate as (R2/R1)^10/(T2/T1)^ where R is the developmental rate and T is the temperature [41]. We used the fraction of larvae emerging as adults to quantify larval survival rather than survival analyses, because larval death included both individuals that were found dead on a known day, and cannibalism, in which larvae disappeared and the date of larval death from cannibalism could not be determined. We used Cox proportional hazard models with Weibull distributions and right-censored data to analyze differences in female adult survival with temperature among populations [42]. We illustrate population response to temperature using the fraction of adult mosquitoes alive 9 days after emergence, which coincides with average lifespan of *Cx. pipiens* in the field for the lowest elevation population [43]. We used generalized linear mixed models with a binomial distribution and a logit link to analyze factors influencing the probability of mosquitoes taking their second blood meal including age, source population, and temperature, as fixed effects, and emergence group, and individual as random effects. For each life history trait, we compared the full fitted model to models that were each missing one fixed effect, by AIC. Finally, we combined the best fitting models for each trait, and models from a previous study [44] to simulate population dynamics. For each population we estimated the number of infectious biting adults (i.e. those taking their second bloodmeal and therefore infectious for WNV) at temperatures 20–35 °C.

## Results

In total, we measured juvenile survival for 9659 individuals (2415 ± 976 (mean ± SD) per population), development time for 4099 females (1025 ± 79), adult mortality for 922 females (230 ± 56), and biting rate for 39 females (10 ± 5). The best fitting models by AIC for larval and adult survival, and development rate were the full models which included population, temperature, temperature^2^, population*temperature, and population*temperature^2^ (Additional file 1: Tables S1–S4). For biting rate, the best fitting model did not include population or population-temperature interactions, but did include temperature, age when taking the first blood meal, and the number of days between bloodmeals (Additional file 1: Table S5).

**Fig. 2:**
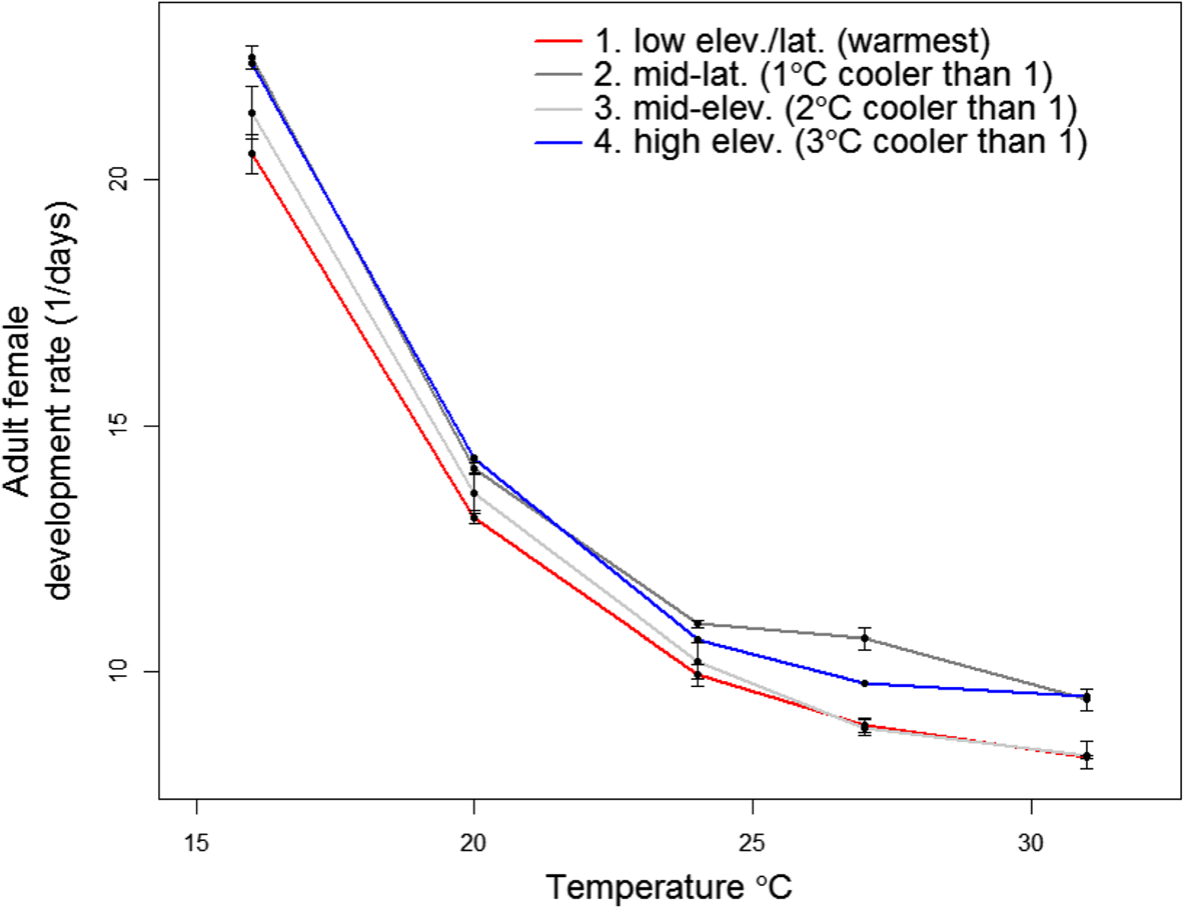
Adult female development time for *Cx. pipiens* (egg hatch to female emergence). All larvae died at 35 °C. *Error bars* show 95 % confidence intervals for replicate flats (200 mosquitoes). For the high population there was only one replicate flat with 200 mosquitoes. Points are jittered along the x-axis to facilitate presentation

Adult female development time decreased monotonically and nonlinearly with increasing temperature, and the greatest decrease occurred at cold temperatures (Fig. 2). A 4 °C increase in temperature from 16 to 20 °C decreased female development time by 57 % (7.8 days) across the four populations, whereas the same 4 °C increase from 27 to 31 °C only decreased development time by 10 % (0.85 days) (Fig. 2). Patterns were similar for adult female development rate – the inverse of development time – which increased at a decelerating rate with increasing temperature (Additional file 1: Figure S3). The Q_10_ temperature coefficient decreased with temperature, from 2.1, between 16 and 27 °C, to 1.5 between 20 and 31 °C.

Variation among populations in developmental time and rate were greatest at extreme temperatures, and smaller than the effects of temperature. The low latitude/altitude population had the fastest development rate across all temperatures. Specifically, at 16 °C the low elevation/latitude population developed 1.9 days (9 %) faster than the high elevation population, and at 31 °C the low elevation/latitude population developed 1.2 days (15 %) faster than the high elevation population (Fig. 2 and Additional file 1: Figure S3).

**Fig. 3:**
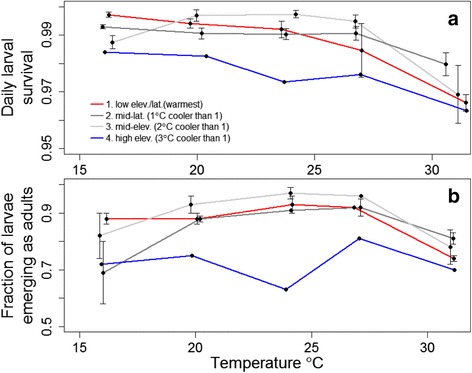
*Cx. pipiens* larval survival. All larvae died at 35 °C. *Error bars* show standard error for replicate flats (200 mosquitoes). For the high population there was only one replicate flat with 200 mosquitoes. Points are jittered along the x-axis to facilitate presentation. **a** Daily juvenile survival. **b** Fraction of larvae that survived to emerge as adults

Average daily larval survival over the developmental period decreased almost linearly with increasing temperature until 31 °C above which it declined more sharply (Fig. 3a). At 35 °C all larvae died before reaching the fourth instar. Overall, a 15 °C increase in temperature from 16 °C to 31 °C tripled daily mortality from 1 to 3 % per day (Fig. 3a). Variation in daily larval survival among populations was substantial but variable across different temperatures. The low elevation/latitude population had the highest larval daily survival (mortality was 6–fold lower than the coolest high-elevation population) at low temperatures (16 °C) but the 2nd lowest survival at 31 °C (mortality was 67 % higher than the mid-latitude population). Additionally, the high elevation population had the highest larval mortality (2.2 times greater than the mid-elevation population).

Larval stage survival (the product of average daily survival and larval development time) increased with increasing temperature at temperatures below 27 °C because development rate increased faster than mortality rate. However, between 31 and 35 °C stage survival decreased sharply because the increase in daily mortality overwhelmed the smaller decrease in developmental rate (Fig. 3b). Across all four populations, a 4 °C rise from 16 to 20 °C increased stage survival by 12 %, whereas stage survival decreased by 16 % from 27 to 31 °C (Fig. 3b). Approximately 67 % of larval mortality was due to larval cannibalism, which showed essentially the same trends as total larval mortality (Additional file 1: Figure S2).

Differences among populations in larval stage mortality were substantial. For example, mortality of the mid-latitude and high elevation populations were twice as high as the low elevation population at 16 °C, and high elevation populations had markedly lower stage survival at most temperatures (Fig. 3). As with patterns across temperatures, these differences among populations in larval stage survival were mostly explained by differences in cannibalism. High elevation larvae were three times more likely to be cannibalized than those from the mid-elevation population, which had the lowest cannibalism and highest overall stage survival across most temperatures (Fig. 3, Additional file 1: Figure S2).

**Fig. 4:**
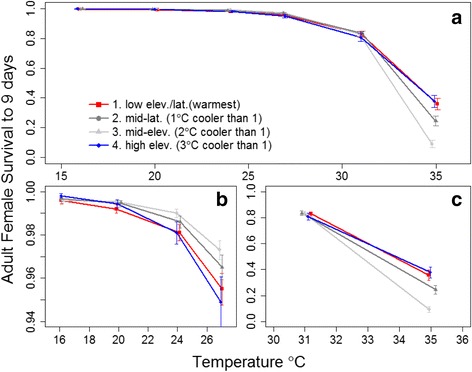
Estimated survival of adult female *Cx. pipiens* to 9 days post-emergence based on Cox proportional-hazard models (see Additional file 1: Figure S1 for raw survival plot). *Error bars* show binomial errors for individual female mosquitoes. Points are jittered along the x-axis to facilitate presentation. **a** Adult female survival from temperature 16-35 °C. **b** Adult female survival at cooler temperatures (16-27 °C). **c** Adult female survival ot warmer temperature (31-35 °C). Note variation in y-axes for **b** and **c**.

Adult female survival decreased nonlinearly and monotonically with increasing temperature, and, as with larval survival, the largest decrease occurred at higher temperatures (Fig. 4, Additional file 1: Figure S4). For all populations, a 4 °C rise, from 16 to 20 °C, resulted in a nearly negligible 0.6 % decrease in female survival, whereas, a similar increase in temperature, from 27 to 31 °C decreased survival 25 % (Fig. 4). Variation among populations was again substantial, with the coldest (high-elevation) population having 2.2 fold lower mortality than the warmest low elevation population at 16 °C (0.19 % vs. 0.42 % daily mortality resulting in average lifespans of 5.3 and 2.4 days, for the high elevation and low elevation populations, respectively). At 27 °C the high elevation population had 88 % higher mortality than the mid-elevation population, but at 35 °C this difference was reversed with the mid-elevation population having a 40 % higher mortality than both the high and low elevation populations which had almost identical survival.

**Fig. 5:**
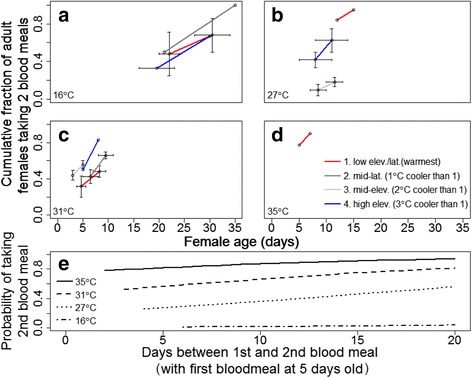
*Cx. pipiens* biting rate. **a**-**d** Cumulative fraction of females taking two blood meals. Each *line* represents a group of females that took their first blood meal on the same day. Each group was then followed over time until they took a second blood meal. A y-value of 100 % means that of the initial females who took their first blood meal on the same day all females within that group went on to take a second blood meal before the study was terminated. *Error bars* are binomial errors based on the number of individuals within a group. **e** Predicted Generalized Linear Mixed-effects Model values for probability of taking a second blood meal, given that age at first blood meal was 5 days old

The cumulative fraction of females taking a second blood meal increased linearly with temperature and the number of days between blood meals, but did not differ significantly among populations, possibly due to small sample sizes (Fig. 5a–d). The effect of age on biting rate was also substantial (Additional file 1: Table S5). For example, at 27 °C, the probability that females would take a second blood meal 7 days later increased from 0.15 to 0.88 as the age when they took their first blood meal increased from 2 to 14 days (Fig. 5e).

**Fig. 6:**
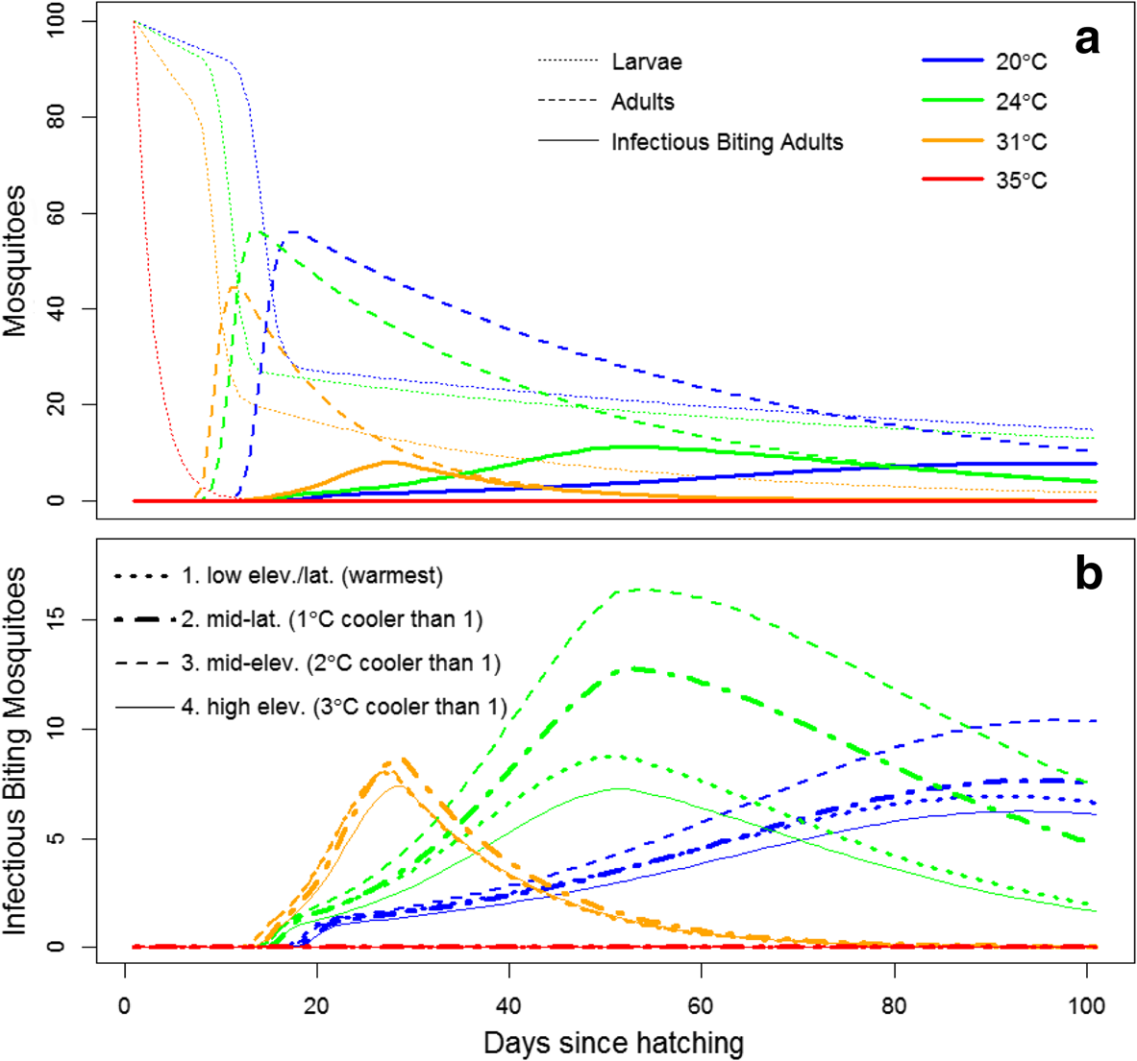
Simulated population dynamics and vectoral capacity for 100 larval *Cx. pipiens* mosquitoes. **a** The effect of temperature on the number of larvae emerging as adults and later becoming infectious biting adults, using the average model coefficients across all populations (Additional file 1: Tables S1–S5), and the relationship between temperature and time and the fraction of mosquitoes transmitting West Nile virus [44]. Each *line color* shows a cohort of 100 mosquitoes over time (hatching on day 0) at a specific temperature and line style indicates the mosquito life stage. Comparison of similar line types indicates the effect of temperature. At 35 °C all larval mosquitoes died before pupation. **b** Variation among populations and temperature on the number of infectious biting adults over time starting from 100 larvae on day 0, as in panel (**a**). *Line color* indicates temperature and line style indicates population. Note the difference in y-axis scales in panels (**a**) and (**b**)

We integrated the best fitting models described above for each trait, and the extrinsic incubation period for West Nile virus [44], to simulate the number of larvae, adults, and infectious biting adults taking their second blood meal over time for each population and temperature (Fig. 6). For all populations, 24 °C produced the highest fraction of infectious biting adults (a peak of 11.3 % of the starting larval population occurring 52 days after hatching; Fig. 6a). At warmer temperatures there were fewer infectious biting mosquitoes, but they were produced earlier (at 24 and 31 °C, infectious mosquitoes peaked at 11.3, and 8.0 % of the starting larval population occurred on days 52 and 28 post-hatching, respectively; Fig. 6a). Differences among populations were very large, and the rank order varied with temperature (Fig. 6b). The mid-elevation population had the highest number of infectious biting adults at 24 °C, which was more than twice as many as the high elevation population at this temperature (Fig. 6b). In contrast, at 31 °C the mid-latitude population had the highest number of infectious biting adults.

## Discussion

Many studies have quantified the effect of temperature on mosquito life-history traits for single populations of a species [45–48]. As in other studies, we found that temperature had strong and relatively consistent effects among populations on development time, larval survival and adult survival, the three most well-measured life history traits, and the patterns were strongly nonlinear. Development time, larval survival, and adult survival decreased with increasing temperature, and biting rate increased. Temperature effects were strongest at higher temperatures for survival and at lower temperatures for development and biting rate.

Although the direction of the relationships we observed between temperature and life history traits are mostly consistent with previously observed patterns, the shape of the temperature performance relationships sometimes differed between our results and previous studies. We found that adult survival decreased monotonically with temperature and female development rate increased monotonically with temperature, whereas some previous studies presented unimodal relationships with temperature [49]. It is worth noting that decreases in life history traits at very high temperatures can be due to rapid death of larval or adult mosquitoes rather than a lack of development or gonotrophic cycling, as we also observed for larval development.

We found significant differences among populations in how life history traits varied with temperature. However, these differences were rarely consistent with a local thermal adaptation hypothesis, which was similar to results from a previous study of two populations of *Culex tarsalis* in California [21]. Some populations of *Cx. pipiens* had uniformly higher or lower performance for some traits across all temperatures, such as the uniformly faster development rate of the warmest population (Fig. 2) or the uniformly lower larval survival of the coldest population (Fig. 3a). The lower larval survival of the highest elevation population may have been influenced by a low number of egg rafts collected from this site. However, the high survival of adults from this population at high and low temperatures demonstrates that not all traits were lower than other populations. In addition, some populations had the worst performance at temperatures where local adaptation would have resulted in them performing the best and vice-versa (e.g. the warmest population in Fig. 3a). Although studies with a larger number of populations or from across a larger spatial temperature gradient might find some evidence for local thermal adaptation, our results suggest that variation that is uncorrelated with local temperatures is substantial and must be incorporated into uncertainty estimates in efforts to predict spatial and temporal variation in disease under climate change scenarios. Our results, and specifically, the magnitude of the site and site-temperature coefficients, provide an estimate of the magnitude of this variation.

## Conclusion

Our results show that the impact of climate change on mosquitoes will be more variable than previous predictions due to the substantial variation that exists in the response of populations to temperature. At the same time, these differences among populations are likely to contribute to spatial variation in transmission, and will be an important source of variation for selection to act on as climate warms [50, 51]. The combination of standing variation and mosquitoes’ evolutionary response will determine the impact of changing climates on vector borne disease.

## Additional file

Additional file 1:
**Mean monthly temperature for each site from 2005-2010.** Data was obtained from the NOAA Climate Data Online site (http://www.ncdc.noaa.gov/cdo-web/). For the high elevation site we calculated the average monthly temperatures using data from the nearby mid-elevation site and the rate of adiabatic cooling with elevation (1.5 °C lower than the mid-elevation site). (DOCX 145 kb)
