# Supplementary material for: Geographic variation in the response of Culex pipiens life history traits to temperature
Source: Parasit Vectors. 2016 Feb 29;9:116. doi: 10.1186/s13071-016-1402-z (PMC4772444; doi:10.1186/s13071-016-1402-z)

**Additional file 1**

**Table S1.** Results from a generalized linear model with a gamma distribution and inverse link of female development time (egg hatch to adult emergence). Each individual mosquito is a data point. The fitted model included population (likelihood ratio test: χ^2^ = 11.49, df = 9, p < 0.001), temperature (linear and quadratic terms) and their interactions (likelihood ratio test: χ^2^ = 445.89, df = 8, p < 0.001).

| Predictor | Coefficient | SE | test stat (t) | P |
| --- | --- | --- | --- | --- |
| Intercept | -0.134 | 0.0050 | -26.94 | < 0.001 |
| Population (Low-elev./lat. (warmest)) |  |  |  |  |
| Mid-latitude (1C cooler than 1) | -0.128 | 0.0046 | -28.12 | < 0.001 |
| Mid-elevation (2C cooler than 1) | -0.126 | 0.0049 | -25.75 | < 0.001 |
| High-elevation (3C cooler than 1) | -0.147 | 0.0091 | -16.10 | < 0.001 |
| Temperature (Low-elev./lat. (warmest)) | 0.015 | 0.00047 | 31.63 | < 0.001 |
| Mid-latitude (1C cooler than 1) | 0.014 | 0.00043 | 33.74 | < 0.001 |
| Mid-elevation (2C cooler than 1) | 0.014 | 0.00046 | 29.83 | < 0.001 |
| High-elevation (3C cooler than 1) | 0.016 | 0.00086 | 18.63 | < 0.001 |
| Temperature^2^ (Low-elev./lat. (warmest)) | -0.00021 | 0.000010 | -20.17 | < 0.001 |
| Mid-latitude (1C cooler than 1) | -0.00022 | 0.0000095 | -23.45 | < 0.001 |
| Mid-elevation (2C cooler than 1) | -0.00018 | 0.000010 | -17.84 | < 0.001 |
| High-elevation (3C cooler than 1) | -0.00025 | 0.000019 | -13.18 | < 0.001 |

**Table S2.** Results from a generalized linear model with a binomial distribution and a logit link of the fraction of larvae emerging as adults. Each individual mosquito is a data point. The fitted model included population (likelihood ratio test: χ^2^ = 312.64, df= 9, p < 0.001), temperature (linear and quadratic terms) and their interactions (likelihood ratio test: χ^2^ = 425.02, df = 8, p < 0.001).

| Predictor | Coefficient | SE | test stat (z) | P |
| --- | --- | --- | --- | --- |
| Intercept | -5.79 | 1.28 | -4.52 | < 0.001 |
| Population (Low-elev./lat. (warmest)) |  |  |  |  |
| Mid-latitude (1C cooler than 1) | -11.19 | 1.12 | -9.96 | < 0.001 |
| Mid-elevation (2C cooler than 1) | -15.87 | 1.46 | -10.86 | < 0.001 |
| High-elevation (3C cooler than 1) | 1.16 | 1.57 | 0.74 | 0.46 |
| Temperature (Low-elev./lat. (warmest)) | 0.75 | 0.11 | 6.61 | < 0.001 |
| Mid-latitude (1C cooler than 1) | 1.11 | 0.10 | 10.89 | < 0.001 |
| Mid-elevation (2C cooler than 1) | 1.65 | 0.13 | 12.34 | < 0.001 |
| High-elevation (3C cooler than 1) | -0.02 | 0.14 | -0.15 | 0.88 |
| Temperature^2^ (Low-elev./lat. (warmest)) | -0.02 | 0.0024 | -7.15 | < 0.001 |
| Mid-latitude (1C cooler than 1) | -0.02 | 0.0022 | -10.39 | < 0.001 |
| Mid-elevation (2C cooler than 1) | -0.04 | 0.0028 | -12.45 | < 0.001 |
| High-elevation (3C cooler than 1) | 0.00 | 0.0029 | 0.16 | 0.88 |

**Table S3.** Results from a generalized linear model with a binomial distribution and a logit link of the fraction of larvae missing due to cannibalism. Each individual mosquito is a data point. The fitted model included population (likelihood ratio test: χ^2^ = 369.35, df= 9, p < 0.001), temperature (linear and quadratic terms) and their interactions (likelihood ratio test: χ^2^ = 420.03, df = 8, p < 0.001).

| Predictor | Coefficient | SE | test stat (z) | P |
| --- | --- | --- | --- | --- |
| Intercept | 4.96 | 1.49 | 3.32 | < 0.001 |
| Population (Low-elev./lat. (warmest)) |  |  |  |  |
| Mid-latitude (1C cooler than 1) | 6.53 | 2.01 | 3.25 | 0.001 |
| Mid-elevation (2C cooler than 1) | 18.01 | 2.70 | 6.65 | < 0.001 |
| High-elevation (3C cooler than 1) | -8.70 | 2.30 | -3.78 | < 0.001 |
| Temperature (Low-elev./lat. (warmest)) | -0.69 | 0.13 | -5.21 | < 0.001 |
| Mid-latitude (1C cooler than 1) | -0.45 | 0.18 | -2.48 | 0.01 |
| Mid-elevation (2C cooler than 1) | -1.67 | 0.25 | -6.75 | < 0.001 |
| High-elevation (3C cooler than 1) | 0.89 | 0.20 | 4.41 | < 0.001 |
| Temperature^2^ (Low-elev./lat. (warmest)) | 0.015 | 0.0028 | 5.50 | < 0.001 |
| Mid-latitude (1C cooler than 1) | 0.007 | 0.0039 | 1.85 | 0.06 |
| Mid-elevation (2C cooler than 1) | 0.035 | 0.0053 | 6.73 | < 0.001 |
| High-elevation (3C cooler than 1) | -0.019 | 0.0043 | -4.52 | < 0.001 |

**Table S4.** Results from a Cox proportional hazard model for the probability of daily female adult survival. Each individual mosquito is a data point. The fitted model included population (likelihood ratio test: χ^2^ = 60.71, df= 9, p < 0.001), temperature (linear and quadratic terms) and their interactions (likelihood ratio test: χ^2^ = 1605.3, df = 9, p < 0.001).

| Predictor | Coefficient | SE | test stat (z) | P |
| --- | --- | --- | --- | --- |
| Population (Low-elev./lat. (warmest)) |  |  |  |  |
| Mid-latitude (1C cooler than 1) | 1.38 | 1.76 | 0.79 | 0.43 |
| Mid-elevation (2C cooler than 1) | 5.57 | 1.67 | 3.35 | 0.001 |
| High-elevation (3C cooler than 1) | -4.53 | 2.49 | -1.82 | 0.069 |
| Temperature (Low-elev./lat. (warmest)) | -0.22 | 0.10 | -2.26 | 0.024 |
| Mid-latitude (1C cooler than 1) | -0.39 | 0.11 | -3.68 | < 0.001 |
| Mid-elevation (2C cooler than 1) | -0.76 | 0.10 | -7.52 | < 0.001 |
| High-elevation (3C cooler than 1) | 0.10 | 0.17 | 0.56 | 0.57 |
| Temperature^2^ (Low-elev./lat. (warmest)) | 0.0089 | 0.0019 | 4.71 | < 0.001 |
| Mid-latitude (1C cooler than 1) | 0.013 | 0.0020 | 6.50 | < 0.001 |
| Mid-elevation (2C cooler than 1) | 0.020 | 0.0019 | 10.50 | < 0.001 |
| High-elevation (3C cooler than 1) | 0.0034 | 0.0032 | 1.085 | 0.28 |
| Blood fed | -2.51 | 0.59 | -4.23 | < 0.001 |

**Table S5.** Results from a generalized linear mixed effects model with a binomial distribution and a logit link for the probability of a female taking a second blood meal. Each individual mosquito was a data point. Individual mosquito and Replicate were included as random effects and the estimated Standard Deviation is given. For all fixed effects the SE of the coefficient is shown.

| Predictor | Coefficient | SE/SD | test stat (z) | P |
| --- | --- | --- | --- | --- |
| Individual mosquito |  | 1.8e-05 |  |  |
| Replicate |  | 0.66 |  |  |
| Intercept | -11.25 | 3.4 | -3.34 | <0.001 |
| Temperature | 0.31 | 0.10 | 3.12 | <0.01 |
| Age at first blood meal | 0.31 | 0.10 | 3.21 | <0.01 |
| Days between bloodmeals | 0.08 | 0.044 | 1.82 | <0.1 |

**Figure S1.** Average mean monthly temperature for each study site over the years 2005-2010. Data was obtained from the NOAA Climate Data Online site (http://www.ncdc.noaa.gov/cdo-web/). Temperature data for the high elevation site was not available on the NOAA website. For this site we calculated the average monthly temperatures using data from the nearby mid-elevation site and the rate of adiabatic cooling with elevation, which suggested that temperatures at the high elevation site would be 1.5°C lower than the mid-elevation site.


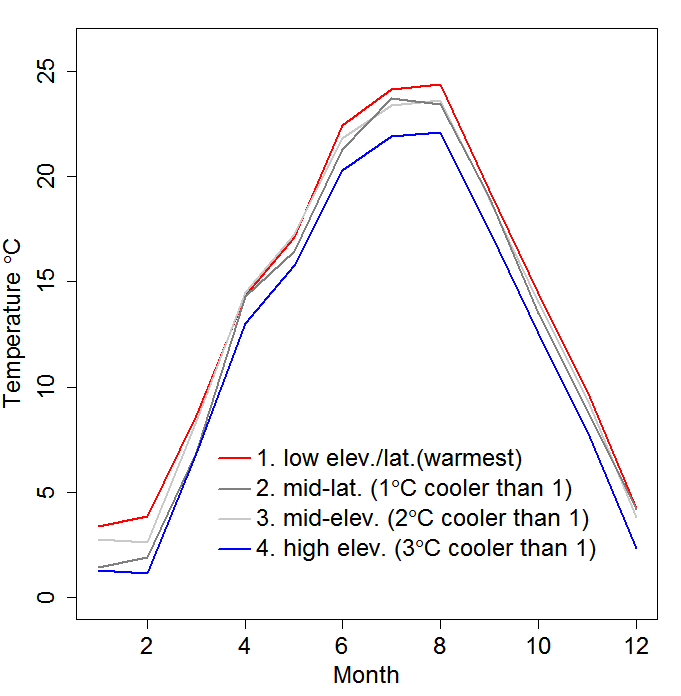


**Figure S2.** Effect of temperature and population on fraction of larvae cannibalized. Error bars show standard error for replicate flats (200 mosquitoes). Points are jittered along the x-axis to facilitate presentation
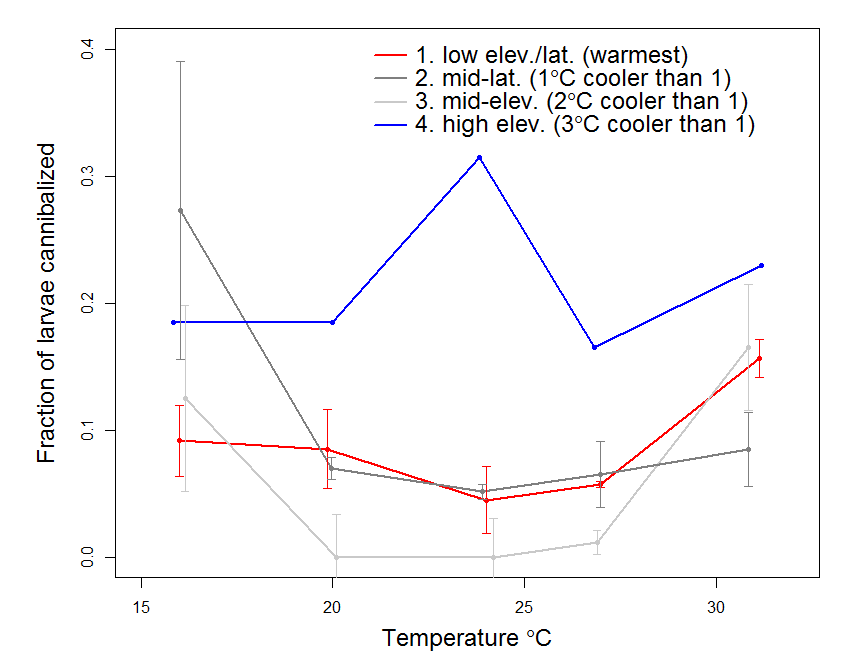


**Figure S3.** Adult female development rate. Error bars show standard deviation for replicate flats (200 mosquitoes). Points are jittered along the x-axis to facilitate presentation.


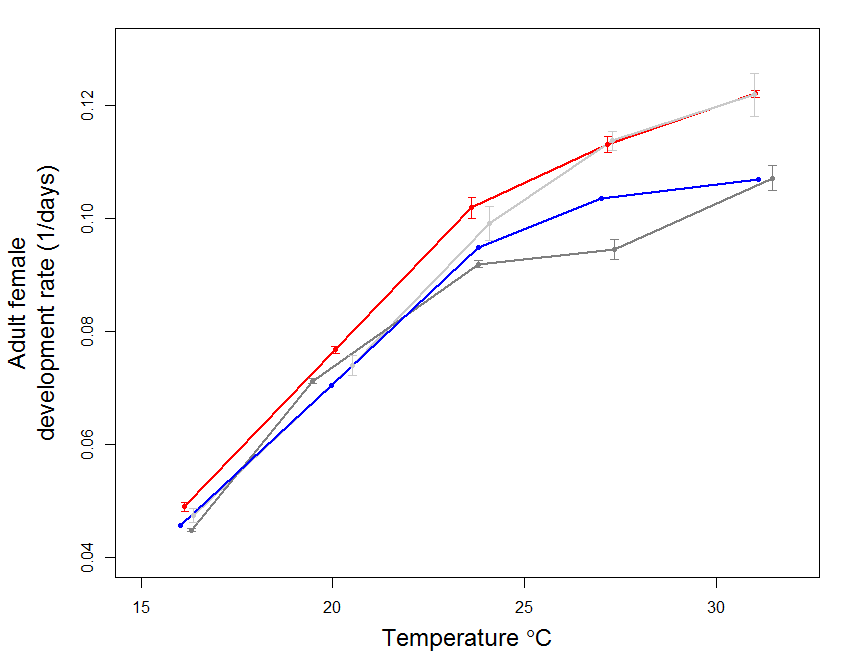


**Figure S4.** Fraction of adult females alive over time (days).


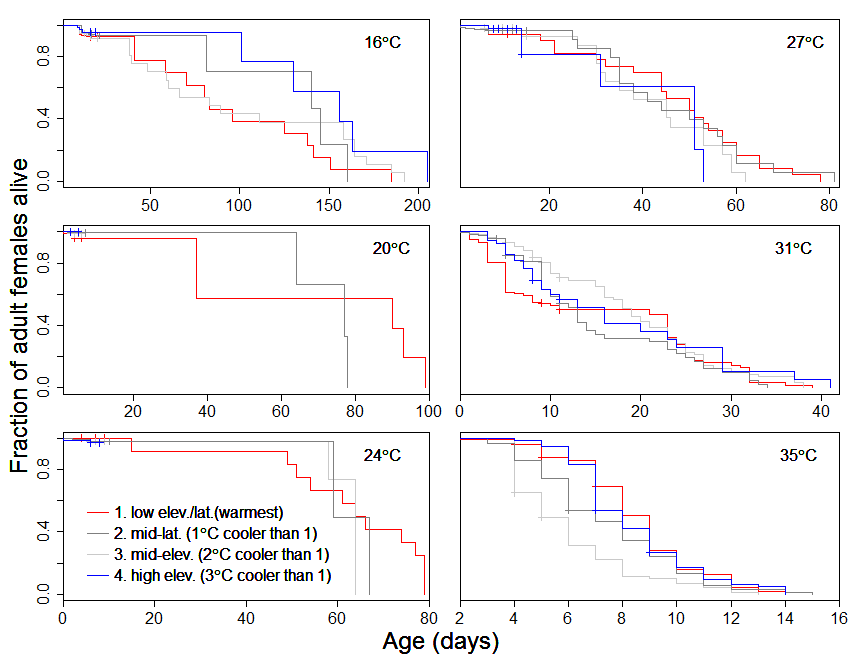

Supplement: Additional file 1: — Mean monthly temperature for each site from 2005-2010. Data was obtained from the NOAA Climate Data Online site (http://www.ncdc.noaa.gov/cdo-web/). For the high elevation site we calculated the average monthly temperatures using data from the nearby mid-elevation site and the rate of adiabatic cooling with elevation (1.5 °C lower than the mid-elevation site). (DOCX 145 kb) [file 13071_2016_1402_MOESM1_ESM.docx]
